# Subclassification of Phenotypic Homozygous Familial Hypercholesterolemia

**DOI:** 10.1016/j.jacasi.2025.06.006

**Published:** 2025-09-02

**Authors:** Hayato Tada, Mariko Harada-Shiba

**Affiliations:** a Department of Cardiovascular Medicine, Kanazawa University Graduate School of Medical Sciences, Kanazawa, Japan; b Cardiovascular Center, Osaka Medical and Pharmaceutical University, Takatsuki, Japan

**Keywords:** familial hypercholesterolemia, genetic diagnosis, low-density lipoprotein cholesterol, low-density lipoprotein receptor, proprotein convertase subtilisin/kexin type 9

## Abstract

Homozygous familial hypercholesterolemia (HoFH) is a rare situation where biallelic genetic disturbance of low-density lipoprotein (LDL) metabolism leads to extreme elevation of LDL cholesterol. There is a great variety of severity in their phenotype, where some patients exhibit premature supravalvular aortic stenosis at their early childhood, whereas others experience myocardial infarction at their adolescence. In addition, there is a set of familial hypercholesterolemia (FH) patients whose phenotype fall into between heterozygous FH and HoFH. Recently, the International Atherosclerosis Society reclassified such patients with FH as “severe FH.” Given that we have several different treatment approaches for these FH patients, including those with HoFH, it is quite important to reclassify them according to their severity of phenotype and types of complications. Here, we propose to clarify so-called “phenotypic HoFH” into 3 groups: severe heterozygous FH, typical HoFH, and severe HoFH based on their LDL cholesterol, genetic backgrounds, frequency, residual LDL receptor activity, and their complications.

## Graphical Abstract

**Figure:**
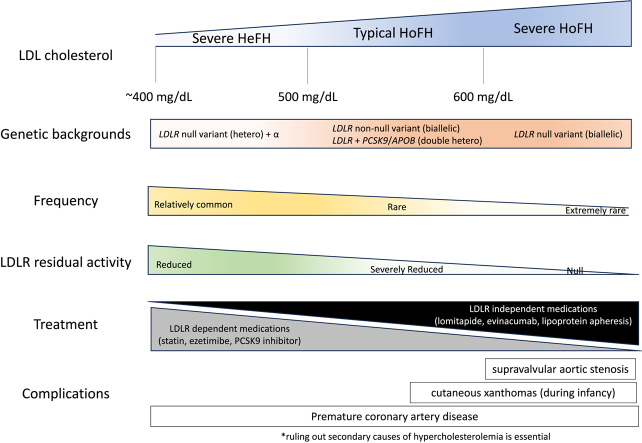

Familial hypercholesterolemia (FH) (OMIM #143890) is a relatively common genetic disorder associated with premature coronary artery disease.^1^ Currently, the prevalence of FH is considered as 1 in 300 general populations.^2^ It is quite important to make early diagnosis of FH to prevent cardiovascular events by appropriate treatment. Among this relatively “common” disorder, there is an important situation that has been described as homozygous familial hypercholesterolemia (HoFH) that is caused by biallelic genetic disturbance of low-density lipoprotein (LDL) metabolism in the narrow sense. Basically, the manifestations of HoFH have been described as a more severe type of heterozygous familial hypercholesterolemia (HeFH) where premature coronary artery disease has been documented as early as their adolescence.^3^ On the other hand, there are some other complications, such as cutaneous xanthomas during their infancy and supravalvular aortic stenosis at their childhood, that are unlikely to be observed in cases with HeFH. In addition, there is a great diversity in their responsiveness to statins and proprotein convertase subtilisin/kexin type 9 (PCSK9) inhibitors among patients with HoFH, which is not the case in patients with HeFH; thus, we need to suspect patients as HoFH when there is a poor response to PCSK9 inhibitors.^4^ Accordingly, it is quite vital to differentiate HoFH from HeFH because of the difference of the appropriate timing and intensity of the treatments. The diagnosis of HoFH has been made phenotypically and/or genetically for a long time, and thus some confusion exists regarding their classification.

## Genetic Aspect of HoFH

Regarding the genetic diagnosis of HoFH, there are several important issues need to be considered. First, the sensitivity of the genetic testing for HeFH has been shown to be around 60%. In other words, as much as 40% of the patients with HeFH do not have any pathogenic FH variants so far. As such, in the cases with HoFH, it is estimated that the majority of the patients with HoFH do not have biallelic pathogenic FH variants. So, the diagnosis of HoFH cannot be ruled out based on the “negative genetic testing results.” Second, it has been shown that the degree of disturbance of LDL metabolism appears to differ according to FH pathogenic variants, leading to their great diversity of their phenotypes. In general, so-called protein-truncating variants in low-density lipoprotein receptor *(LDLR)* lead to more severe phenotypes compared with missense variants in *LDLR*. In addition, there are at least 4 different genes that are considered a “FH-gene,” including *LDLR*, apolipoprotein B (*APOB*), *PCSK9*, and low-density lipoprotein receptor adaptor protein 1 (*LDLRAP1*). It has been shown that the degree of disturbance by *LDLR* variants to LDL metabolism appear to be larger than those by others. These factors should contribute to their great diversity of their phenotypes. Third, there are at least several genetic factors, such as rare variants of ATP binding cassette subfamily G member 5 (*ABCG5*), ATP binding cassette subfamily G member 8 (*ABCG8*), and apolipoprotein E (*APOE*), that are contributing to worsen their phenotypes of HeFH, making it difficult to differentiate them from patients with HoFH. In fact, we previously named them as oligogenic FH in contrast to the simple HeFH.^5^

## The Emerging Concept of Severe HoFH

In addition to these genetic aspects, the International Atherosclerosis Society has proposed the concept of “severe FH” to identify the patients with FH whose cardiovascular risk appears to be even higher.^6^ The definition of “severe FH” does not include the genetic aspect itself; however, this concept in the broad sense should include HeFH and HoFH as well in terms of genetic backgrounds. As stated previously, it is important to differentiate HoFH from HeFH because of the following: 1) novel therapies whose mechanisms are independent of LDLR pathway are available in patients with HoFH; 2) there are some complications that are quite specific and in patients with HoFH; and 3) LDL-lowering therapies, including the ones stated previously, need to be introduced as soon as possible in patients with HoFH, compared with the situation where LDL-lowering therapies are recommended to be introduced at age 8 to 10 years in patients with HeFH. In addition to discrimination of HoFH from HeFH, we now present the emerging concept of “severe HoFH,” in contrast to the “typical HoFH.” It is true that HoFH in general is the special situation where LDL-lowering therapies need to be introduced as early as possible because of their extremely high risk of premature coronary artery disease, but another severe complication, supravalvular aortic stenosis, in a portion of patients with HoFH. Supravalvular aortic stenosis associated with calcified aorta typically starts to develop during infancy to childhood in some patients with HoFH. This specific situation is quite different from the common complication of premature coronary artery disease in terms of treatment. This situation requires very high-risk surgical operation of aortic valve replacement as well as a reconstructive procedure to the aortic root to ascending aorta. Accordingly, we need to identify such patients with HoFH who need to start LDL-lowering therapies during their infancy. Moreover, liver transplantation may be an option for such patients under some specific circumstances.^7^

## Genetic Testing for FH in Patients With Severe Hypercholesterolemia

Genetic testing for FH in patients with severe hypercholesterolemia has several important roles: 1) definitive diagnosis as FH regardless of zygosity; 2) discrimination among heterozygous, homozygous, and others; 3) enhancement of cascade screening; and 4) risk stratification and treatment adjustments based on their genetic backgrounds. As stated previously, it is important to recognize that the sensitivity of the genetic testing for FH is ∼60%, and thus, some patients with severe hypercholesterolemia may not have any identifiable pathogenic variants associated with FH, whereas others may carry variants of uncertain significance. In those situations, classification based on phenotypic characteristics would be necessary. In addition, response to LDL cholesterol-lowering treatment might provide clues to distinguish between those with HeFH and HoFH

## Classification of Severe HeFH, Typical HoFH, and Severe HoFH

**Figure 1:**
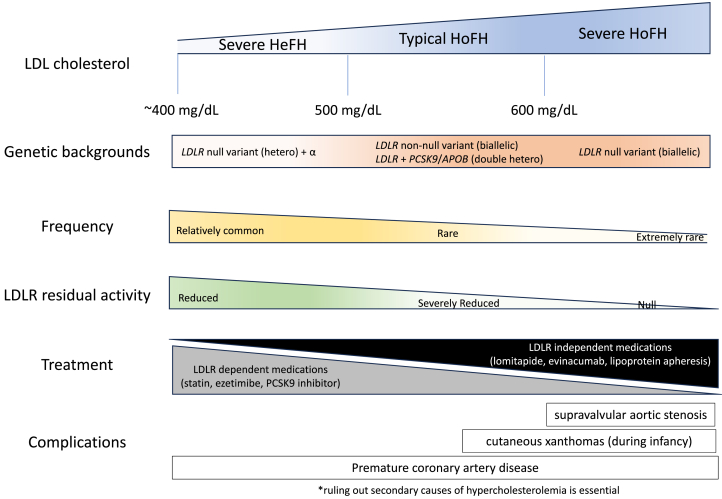
The Concept of Severe HeFH, Typical HoFH, and Severe HoFH Classification of severe heterozygous familial hypercholesterolemia (HeFH), typical homozygous familial hypercholesterolemia (HoFH), and severe HoFH according to low-density lipoprotein (LDL) cholesterol, genetic backgrounds, frequency, residual low-density lipoprotein receptor (LDLR) activity, their complications, and treatment are provided.

Now, we are providing a simple concept of classification of severe HeFH, typical HoFH, and severe HoFH according to LDL cholesterol, genetic backgrounds, frequency, residual LDLR activity, their complications, and treatment (Figure 1). In general, the situation of severe HeFH is caused by heterozygous *LDLR* null variant and other LDL-raising genetic variant(s) with moderate effect size such as *ABCG5*/*ABCG8*/*APOE*. It is important to note that they never exhibit cutaneous xanthomas during infancy or supravalvular aortic stenosis during their childhood. The situation of typical HoFH is caused by biallelic non-null *LDLR* variants or double heterozygous of *LDLR* and *PCSK9*. Some of them exhibit cutaneous xanthomas during infancy; however, they rarely exhibit supravalvular aortic stenosis during their childhood. It is important to note that statins and PCSK9 inhibitors are typically effective if not perfectly effective to reduce their LDL cholesterol because their LDLR residual activity usually is not zero. Finally, the situation of severe HoFH is caused by the biallelic *LDLR* null variants where LDLR residual activity is almost zero. They typically exhibit cutaneous xanthomas during infancy and supravalvular aortic stenosis during their childhood. Accordingly, LDL-lowering therapies should be introduced as early as possible during their infancy. We have reported a case of a patient with severe HoFH where statin and ezetimibe were introduced during his infancy; however, supravalvular aortic stenosis had developed at the age of 4 years probably because of the minimal effects of LDL lowering by statin and ezetimibe. Sixteen years later, his LDL cholesterol is controlled below 100 mg/dL under the combination of LDL-lowering therapies of statin, ezetimibe, lomitapide, and lipoprotein apheresis, but his supravalvular aortic stenosis needs to be monitored regularly. We did our best to reduce his LDL cholesterol at that time with statin and ezetimibe; however, novel therapies that are independent of LDLR should have been introduced to him if only they were available at that time, including lipoprotein apheresis.^8^^,^^9^ Another important factor that could attenuate their phenotype is lipoprotein(a). Patients with HoFH appear to exhibit elevated serum lipoprotein(a), which should be associated with increased risk for aortic diseases.^10^ In summary, our reclassification offers several advantages: 1) identifying patients with severe HeFH where PCSK9 inhibitor should be very effective to reduce their LDL cholesterol; 2) clarifying that the presence of cutaneous xanthomas during infancy is the specific phenotype as HoFH (typical of severe); and 3) identifying the most severe patients complicated with supravalvular aortic stenosis in their childhood where the most intensive treatments should be considered during their infancy. We firmly believe that this classification will help us to understand the phenotypic variations among phenotypic HoFH, leading to the best treatment approach that is currently available.

### Study limitations

This study is based on a limited number of cases of HoFH because of the rarity of this disease. However, we experienced and examined hundreds of cases of HoFH so far, leading to this idea.

### Ethical approval

This study was approved by the Ethics Committee of Kanazawa University. All procedures followed were in accordance with the laws and ethical standards of the responsible committee on human experimentation (institutional and national) and with the Helsinki Declaration of 1975, as revised in 2008.

## Funding Support and Author Disclosures

This work has been supported by a scientific research grant from Health, Labour and Welfare Sciences Research Grant for Research on Rare and Intractable Diseases. The authors have reported that they have no relationships relevant to the contents of this paper to disclose.
